# Effects of laser treatments on surface roughness of zirconium oxide ceramics

**DOI:** 10.1186/s12903-018-0688-y

**Published:** 2018-12-19

**Authors:** Goknil Ergun Kunt, Ibrahim Duran

**Affiliations:** Department of Prosthodontics, Faculty of Dentistry, Ondokuz Mayis University, 55139, Atakum, Samsun, Turkey

**Keywords:** Y-TZP, Er: YAG laser, CO_2_ laser, Surface roughness, Profilometry, SEM, Sandblasting

## Abstract

**Background:**

The aim of this study was to analyze the surface roughness of yttrium stabilized tetragonal polycrystalline zirconia (Y-TZP) ceramics after different laser treatments (CO_2,_ ER: YAG).

**Methods:**

5x5x2 mm rectangular prisms of forty eight Y-TZP (Zirkonzahn) ceramic specimens were prepared. In order to standardize surfaces, 600-, 1200- grid silicon carbide papers were used to gradually ground wet on 300 rpm grinding machine for 10 s. Eight groups (*n* = 6) were randomly formed from the specimens of each ceramic as control (GroupC), sandblasted (GroupS), two different CO_2_ laser treatments (Group3W: 3 W and 382 w/cal, Group4W: 4 W and 509w/cal) and four different Er: YAG laser treatments (Group150SP: 150 mJ and 10-Hz with 100μS; Group150SSP: 150 mJ and 10-Hz with 300μS; Group300SP: 300 mJ and 10-Hz with 100μS; Group300SSP: 300 mJ and 10-Hz with 300μS). A profilometer was used to conduct surface roughness measurements (Ra). Surface morphologies of the specimens were evaluated under SEM after laser treatment.

**Results:**

To analyze the data one-way ANOVA and to compare the mean values Tukey HSD tests (α = .05) were used. One - way ANOVA results showed that Group S had the highest Ra value and Group150 SP had the lowest. After sandblasting group the highest value was seen in Group4W. There were no statistically significant differences among Group C, Group3W, Group150SSP, Group300SP, and Group300SSP.

**Conclusions:**

The study showed that surface roughness of zirconium oxide ceramics was increased with CO2 laser.

## Background

Recently, one of the most frequently used all ceramic core material for fixed restorations (crown and bridge), orthodontic brackets and CAD/CAM technology is yttrium-stabilized- tetragonal-zirconia-polycrystal (Y-TZP) [1]. It has many advantages like high aesthetic profile, biocompatibility, chemical stability and exceptional mechanical features including hardness, 700–1200 MPa high flexural strength and 7–10 MPa m ½ fracture toughness [2, 3].

A strong and solid bond between cement and zirconia is extremely important for patients’ satisfaction. Marginal seal, proper retention and sufficient aesthetics are improved characteristics of resin cements over conventional cements [4, 5]. However, for sufficient bonding one of the vital components is micromechanical attachment [5–7]. Roughening zirconia restorations inner surfaces causes increases in the area convenient for penetration and in situ polymerization of resin based materials, which in turn enhance the mechanical bond.

There are many surface treatment methods to improve a succesful bonding. Hydrofluoric acid etching, which is one of the most effective methods to increase the bonding mechanism, is not a useful technique due to the fact that the zirconia is not glassy and is densely-sintered. Studies to ensure a good bonding in zirconia have been shown for many years that the surface should be cleaned first and then roughened. Then chemical activations such as airborne particle abrasion using pure alumina or silica coating using silica-coated alumina particles can be carried out. Due to the improvement of lasers in dentistry laser irradiation is thought to be an alternative method to increase surface roughness and improve adhesion between ceramics and resin cements [8–15].

Recently, for different dentistry practices including cavity preparation [16], carious dentin removal, surface conditioning [17–19] and also as a surface treatment of indirect restorations [20], erbium:yttrium aluminum garnet (Er: YAG) laser is recommended. Because of the synchronization of its wavelength and the main absorption peak of water and because of its good absorption by OH^−^ groups in hydroxyapatite, it is frequently used on dental ceramics [8]. The carbon dioxide laser (CO2) is commonly used intraorally especially in soft tissue and hard tissue applications [21, 22]. Because ceramic nearly totally absorbs CO2 laser wavelength, CO2 laser is very suitable for the surface treatment of ceramic materials [23]. CO_2_ laser etching may represent an effective method for conditioning zirconia surfaces, enhancing micromechanical retention and improving the bond strength [24]. Conchoidal tears, which result from surface warming, occur with the heat initiation of surfaces of ceramic by focusing CO2 laser. These tears are believed to supply mechanical success between resin composite and ceramics retention [25–27].

In order to prevent damage to the zirconia surface, laser settings like pulse, power and duration have great importance. In order to strengthen bonding and durability of the restorations, all the above mentioned procedures stress increasing the surface area [24]. Despite the fact that various surface treatments have been proposed, it is still a challenge to choose the most appropriate method. The purpose of this study was to examine the differences and similarities of surface roughness of untreated and sandblasted and laser applicated surfaces of zirconia. The research hypothesis was that different energy values of Er: YAG and CO_2_ laser treatments affect surface roughness of the zirconia.

## Methods

**Fig. 1:**
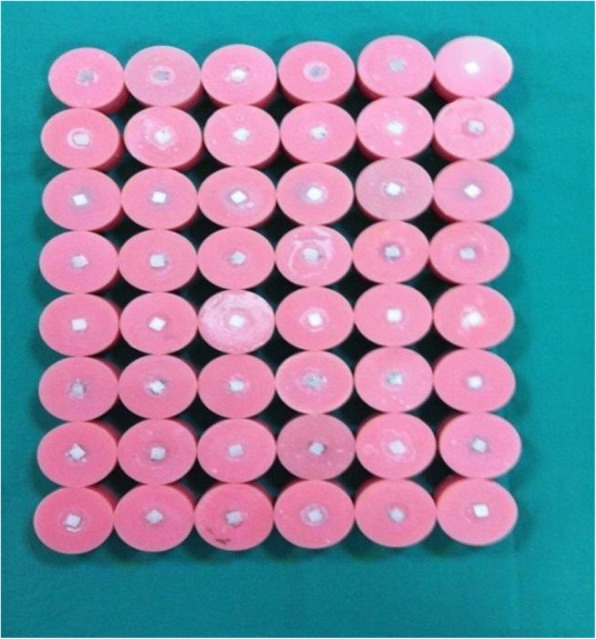
Forty-eight yitriyum stabilized tetragonal polycryistalin zirconia ceramic specimens were prepared

In the study 5x5x2 mm of rectangular prism of forty eight zirconia core specimens were produced by a copy-milling system (Zirconzahn, Bruneck, Italy) using prefabricated blanks of zirconia (ICE Zircon Translucent; Zirconzahn) and then sintered according to the manufacturer’s instructions. The specimens’surfaces were firstly cleaned with ethanol and then air-dried before surface treatment (Branson 2210; Branson Ultrasonics Corporation, Danbury, CT). Zirconia cores were embedded in the centers of autopolymerizing acrylic resin blocks (Meliodent; Heraeus Kulzer, Armonk, NY). In order to standardize surfaces, 600-, 1200- grid silicon carbide papers (English abrasives, English abrasives Ltd. England) were gradually grounded using water coolant on a 300 rpm grinding machine for 10 s (Beuhler Metaserv, Germanyand ultrasonically cleaned for 3 min in ethanol and deionized water and then air-dried. Subsequently, specimens were randomly divided into eight groups, each containing 6 specimens, for the following different surface treatment methods (Fig. 1), (Table 1).

- **GroupC:** Control group, no treatment.
- **GroupS:** Sandblasted specimens with 50-μm aluminium oxide powder (Korox 50, Bego, Bremen, Germany) at 2.8 bars from a 10 mm distance for 15 s. After sandblasting, in order to remove the remaining powder, compressed air was used to clean the specimens.

**Table 1:** Groups and surface treatments

Groups	Surface Treatments
Group C	Control group, specimens which were untreated.
Group S	Specimens were sandblasted with 50-μm Al_2_O_3_ powder (Korox 50, Bego, Germany) at 2.8 bar for 15 s through a nozzle distance of 10 mm.
Group 3 W	CO_2_ laser: the applied energy level was 382w/cal 3 W.
Group 4 W	CO_2_ laser: the applied energy level was 509w/cal 4 W.
Group 150SP	Er:YAG laser: the applied energy level was 150 mJ with 10-Hz frequency for 45 s. The pulse width 100 μS.
Group 150SSP	Er:YAG laser: the applied energy level was 150 mJ with 10-Hz frequency for 45 s. The pulse width 300 μS.
Group 300SP	Er:YAG laser: the applied energy level was 150 mJ with 10-Hz frequency for 45 s. The pulse width 300 μS.
Group 300SSP	Er:YAG laser: the applied energy level was 300 mJ with 10-Hz frequency for 45 s. The pulse width 300 μS.

### CO_2_ laser treatments

**Fig. 2:**
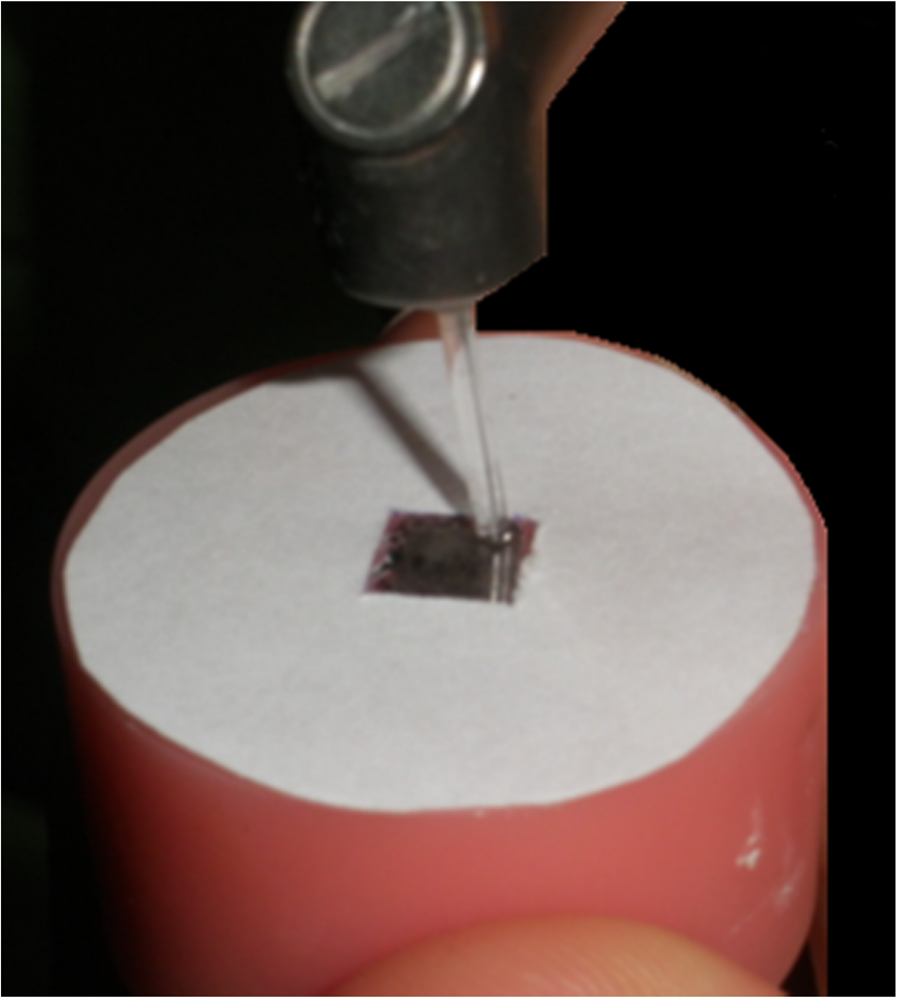
The application tip was moved from bottom to top and maintained in slight contact with the zirconia surface

The specimens were treated by using CO_2_ laser (Smart US-20D, DEKA, Firenze, Italy) working at 10.6 μm. 3 W- 4 W energy level was applied at a continuous and non-contact mode. The application tip’s diameter was 1 mm and its length was 12 mm. Moving up and down, zirconia surfaces were processed with the application tip in slight contact [28, 29] (Fig. 2). The applied energy levels were:

- **Group 3W:** The applied energy level was 382w/cal and 3W
- **Group 4W:** The applied energy level was 509w/cal and 4W

### Er YAG laser treatments

The specimens were treated by using Er: YAG laser (Fotona AT Fidelis, Ljubljana, Slovenia) at 2940 nm. A 90°-angled dental hand instrument (R14-C, Fotona) with a sapphire cylindrical (1.3 × 12 mm) fiber-optic tip was used at an incidence angle of 90° with water irrigation. Air pressure and also water pressure were fixed at 2 bars. Moving up and down, zirconia surfaces were processed with the application tip in slight contact [28, 29] . The applied energy levels are given below:

- **Group150SP:** The applied energy level was 150 mJ with 10hz frequency for 45 s. The width of the pulse was 100 μS.
- **Group150SSP:** The applied energy level was 150 mJ with 10hz frequency for 45 s. The width of the pulse was 300 μS.
- **Group300SP:** The applied energy level was 300 mJ with 10hz frequency for 45 s. The width of the pulse was 100 μS.
- **Group300SSP:** The applied energy level was 300 mJ with 10hz frequency for 45 s. The width of the pulse was 300 μS.

All of the specimens were fixed into a brass mold by using silicone impression material and on each sample a profilometer was used to asses three roughness measurements (Ra, μm) (Perthometer M2, Mahr GmbH, Göttingen, Germany). A cut-off value of 0.25 mm was found, which allowed the detection of only those irregularities. A diamond stylus (NHT-6), which had a radius of 2 μm and 90° angle, was transversed with a force of 0.7 N at a constant speed opposite each of the finished samples. The profilometer was calibrated before measurements of each group. Care was taken to make profilometer records as close as possible to the sample center. The mean of the surface roughness measurements was evaluated to find out the samples’ surface properties. Ra is an average value which gives information about the surfaces traced by the profilometer. When this value (Ra) is low it means smoother surface.

One-way analysis of variance (ANOVA) and post-hoc multiple comparisons Tukey’s tests were used to analyze the surface roughness measurements (Ra values) at a significance level of *p* < 0.01 by using statistical software program (SPSS for Windows, Version 12.0.1; SPSS Inc., Chicago, IL, USA).

For the assessment of surface morphology, the samples used profilometry were coated in gold-palladium (Quorum Technologies Polaron SC7620, Newhaven, East Sussex, UK) and observed under SEM (JSM-6610 LV Scanning electron microscope, JEOL USA) with 50X, 100X, and 500X magnifications and 15 kV voltage.

## Results

**Fig. 3:**
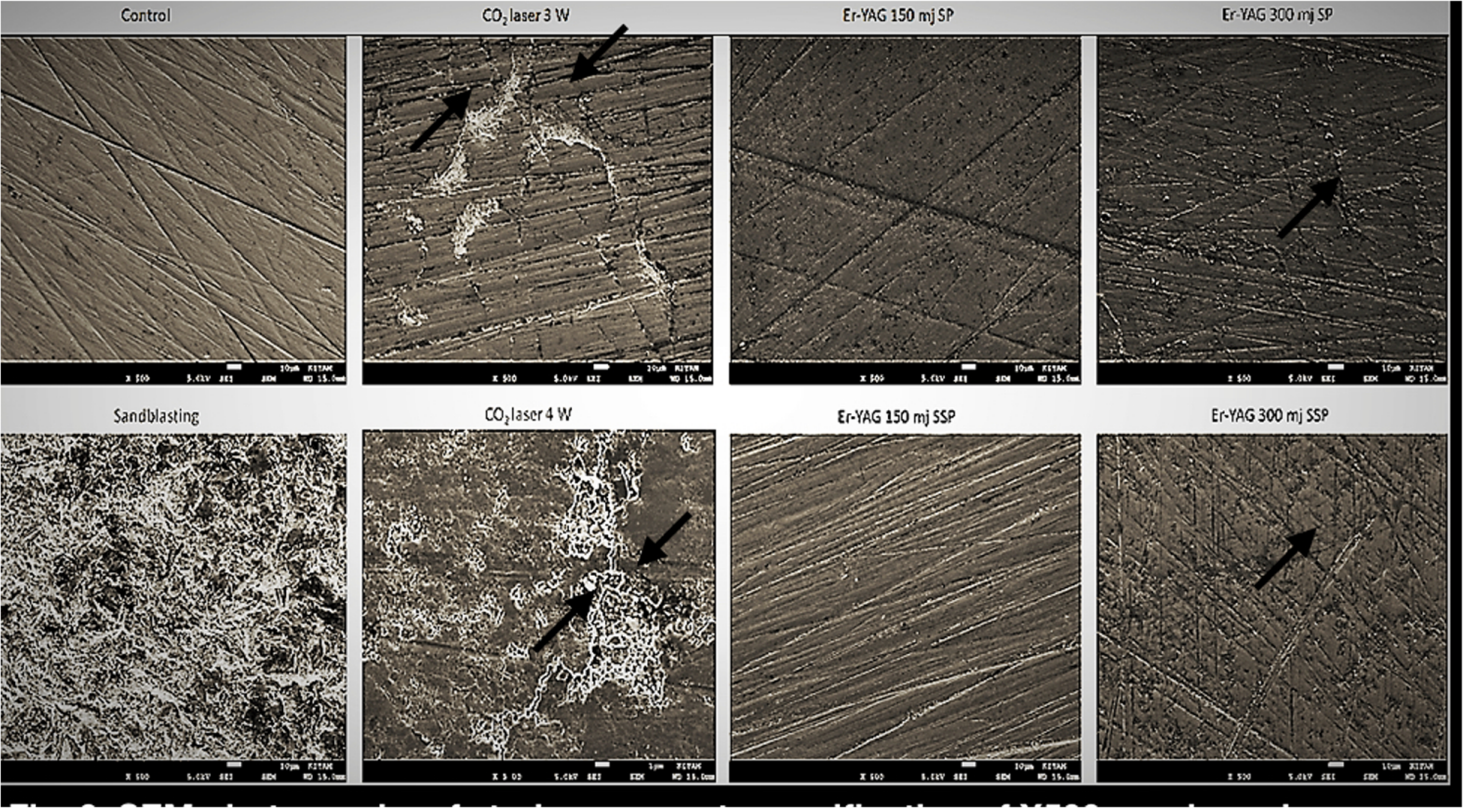
SEM photographs of study groups at magnification of X500 roughened areas for CO_2_ and micro cracks for ER-YAG laser were shown with arrows

Statistical analyses of surface roughness values treated with different groups found are presented in Table 2. ANOVA test and also Tukey’s test (α = 0.05) showed that all treatments yielded different mean surface roughness values. The highest Ra value was showed in Sandblasting group (GroupS = .876 ± .067). After it CO_2_ laser treatment group followed (Group4W = .622 ± .177) it. There were statistical differences between the two groups (*p* < .001). Also there were statistically significant differences rest of the groups. There were no statistically significant differences among control (GroupC), CO_2_ laser treatment (Group3W) and Er YAG laser treatments (Group150SP, Group150SSP, Group300SP, Group300SSP). Similar morphologic differences were seen between the specimens’ surfaces after different surface treatments in SEM images. When compared with other treatments, irregular surface pattern on Y-TZP surfaces were found only in sandblasting with 50 mμ Al_2_O_3_ particles. Perceptible loss of material was caused by Er: YAG laser irradiations and smooth areas confined by small fissures and narrow microcracks on the surfaces appeared due to both energy intensities. SEM examination showed softening, too much loss of mass and existence of deep cracks in these groups (Fig. 3). Only 4 W CO_2_ laser irradiation caused scaly irregularities on the Y-TZP surfaces. An increase on surface roughness was seen as a result of treatment of the surface of Y-TZP discs with CO_2_ laser as a result of comparison with Er: YAG laser (*p* < 0.001) and this result was thought to occur because of the degree and kind of surface irregularities produced on the Y-TZP surface.

**Table 2:** Mean surface roughness (Ra) values and standard deviations (SD) of the groups

Groups	N	Mean Ra (μm)	SD	*P*
Group C	6	0.102^c^	,034	*p* > .001
Group S	6	0.876^a^	,067	*p* < .001
Group 150SP	6	0.102^c^	,023	*p* > .001
Group 150SSP	6	0.140^c^	,035	*p* > .001
Group 300SP	6	0.110^c^	,032	*p* > .001
Group 300SSP	6	0.121^c^	,021	*p* > .001
Group 3 W	6	0.158^c^	,030	*p* > .001
Group 4 W	6	0.622^b^	,177	*p* < .001
Total	48	0.279	,290	

Different letters indicate statistically difference (*p*<.001)

## Discussion

The purpose of this study was to investigate the effect of air abrasion with Al_2_O_3_ and different outputs and energy levels of Er: YAG and CO_2_ laser applications on the surface roughness of Y-TZP. Since the tested treatments changed the surface roughness of Y-TZP, it can be said that the null hypothesis could be accepted based on the results of this investigation.

There are a great number of extraoral methods to obtain a robust and permanent bond between the tooth and restoration like sandblasting and mechanical abrasion [30, 31]. While creating micro cracks in zirconia to increase retention also it weakens mechanical properties of zirconia [32, 33]. Therefore, recently other methods such as laser etching have been introduced to create surface roughening and for the enhancement of zirconia–veneer ceramic interfacial bonding and integration this method might prove to be a new method of surface treatment [34]. It should be kept in mind that surface alterations may occur on the zirconia surface due to laser irradiation.

In many studies air abrasion is the most effective surface treatment method than the application of CO_2_ and Er:YAG lasers and it has been shown that irradiation of Y-TZP ceramic surfaces with CO_2_ and Er:YAG lasers did not increase the surface roughness significantly [25, 30, 35–37]. Conditioning with air-abrasion can cause resin-ceramic bonding through improving surface roughness and bonding surface area [38]. Demir et al. [35] stated that in order to get micromechanical retention before luting, air abrasion or 400 mJ Er: YAG laser energy can be used; however, air abrasion was found be the most useful surface treatment method since it had significantly higher surface roughness values than the control group and different modes of Er: YAG (200, 300, and 400 mJ). In their study they analyzed Y-TZP disks after irradiation of various power settings with Er: YAG, CO_2_ or diode laser, Stübinger et al. [36] found diode lasers to be the best system giving surface preservation and safety in the treatment of zirconia implant surface. In their study they analyzed different Er: YAG laser energy intensities to find out their influences on surface roughness and morphologic characteristics of Y-TZP, Cavalcanti et al. [27] concluded that excessive material deterioration occurred as a result of greater laser modes (400 and 600 mJ), making them unfit as surface treatments for zirconia surfaces. In a study by Miranda et al. [37], which examined the surface roughness on Y-TZP surface after Er: YAG laser irradiation at 1.5 W/20 hz concluded that laser irradiation caused a decrease on surface roughness. The present study used maximum 300 mJ output power of the Er: YAG laser with various pulse widths and it was found that it did not roughen the Y-TZP surfaces to accept a surface treatment method. Miranda et al. [38] evaluated the surface roughness on Y-TZP surface after Er, Cr: YSGG laser irradiation at 1.5 W/20 Hz and found that laser irradiation decreased the surface roughness.

Ersu et al. [21] compared and assessed the results of CO_2_ laser and conventional surface treatments on surface roughness of in-ceram zirconia discs to dentin and found that for roughening surfaces of the specimens, sandblasting was an effective surface treatment and surface roughness was not increased by CO_2_ laser irradiation.

In contrast to above studies, irradiation of zirconia ceramic surfaces with CO_2_ and Er:YAG lasers resulted with an increase on surface roughness in many studies [31, 39–41]

Untreated, sandblasting, laser irradiations (Er: YAG and Nd: YAG laser) and mixtures of these laser applications with sandblasting on pre-sintered ZrO_2_ was examined by Kirmali et al. [39] and they concluded that the surface roughness values increased significantly through sandblasting and Er: YAG laser applications on pre-sintered ZrO2 substructures.

Liu et al. [40] performed X-ray diffractometric analysis of zirconia ceramic samples after sandblasting and CO_2_ laser application. In parallel with this study, both sandblasting and CO2 laser irradiation were found to raise the surface roughness of zirconia specimens, while the laser group had lower increase than the sandblasting group.

The results of various lasers and particle abrasion on surface characteristics of zirconia ceramics were examined by Arami et al. [41] and all treated surfaces were found to have higher roughness than the control group. Similar surface roughness was found in surfaces treated by Er: YAG laser and air abrasion showing that this laser can be a suitable substitute for air abrasion.

In order to find out the reliable intensity for roughening the Y-TZP surface, different energy intensities were compared in a great number of studies. The laser energy settings in our study were chosen according to previous study reports and the effectiveness of these parameters was examined [23–29]. The methodology for laser application on ceramic specimens is based upon a series of pilot studies. However, there is a need for further studies since there is still no clear information about effective laser type and applications modes.

Y-TZP’s surface roughness was described by Ra parameters found with a profilometer. The overall roughness of a surface is described by this parameter and it is the arithmetical average value of all absolute distances of the roughness profile from the center line within the measuring length. Surface roughness (Ra) is the finer irregularities of the surface texture caused by the action of the production process or material condition and is expressed in micrometers (μm).

Besides the type of irradiated ceramic, the range of superficial changes on Y-TZP ceramic surface is based on the energy density of the laser radiation. The main effect of laser energy is the conversion of light energy into heat, with the most significant between the laser and substrate being the absorption of the laser energy by the substrate.

In addition to other surface qualities, pigmentation of the surface and its water content determines the extent of energy absorbed by the irradiated surface. Since Y-TZP ceramic is white opaque and there is no water content, retention of laser energy is difficult on Y-TZP ceramic. In this study, graphite powder is used to raise retention of laser energy by zirconia in all laser groups due to this difficulty. Sandblasted specimens showed significant higher surface roughness than the others despite this method among all groups. Higher surface roughness measurements when compared with other laser treated groups were found only in 4 W CO_2_ laser irradiated Y-TZP groups and statistically significant differences were found (*p* < .05). Group C, Group 3 W, Group 150SP, Group 150SSP, Group 300 SP, and Group 300 SSP did not show statistically different surface roughness measurements (*p* > .05). An uneven surface with a rough characteristic is formed by applying Er YAG laser and the micro cracks found in electron microscope evaluation confirm our results (Fig. 3).

According to the results of our study, the most effective treatment to roughen the surface of Y-TZP ceramics was found to be sandblasting. Different Er Yag and CO_2_ laser irradiations did not roughen the Y-TZP ceramics. Surface roughness measurements after laser irradiations were almost the same with those of the control group.

## Conclusions

The materials used in our study gave different surface roughness, the highest surface roughness values were found with sandblasting. Nevertheless, there were no significant differences in surface roughness between laser treated specimens and the control, except 4 W CO_2_ laser treatment. As an alternative surface treatment to sandblasting for Y-TZP ceramics, only 4 W CO_2_ (Group 4 W) laser irradiation technique is recommended (*p* < .05).

## Abbreviations

ANOVA: One-way analysis of variance
CAD CAM: Computer aided design computer aided manufacturing
CO2: Carbon dioxide laser
Er YAG: Erbium yttrium aluminum garnet
Ra: Roughness measurements
Y-TZP: Yttrium stabilized tetragonal polycrystalline zirconia
